# Should Medical Practitioners Dispense?

**Published:** 1910-06-25

**Authors:** 


### SPECIAL ARTICLE.

### SHOULD MEDICAL PRACTITIONERS DISPENSE?

[*The question which heads this article is one that is arousing considerable attention at the present moment, and in the circumstances the opinions of two practical men, looking at the subject from opposite standpoints, are of considerable interest. We shall be glad to receive further communications from practitioners upon the question.—Ed.* The Hospital.]

### I.—THE PROFESSIONAL STANDPOINT.

By A HOSPITAL PHYSICIAN.

One of the many results of specialisation has been separation into two separate groups—pharmacists or those who prepare drugs for use, and medical men who advise what particular drugs should be employed in a given case. Circumstances, however, here as in other special departments of medicine, make it impossible for the line of demarcation to be in any way a sharp one. Hence it results that, while there are medical men who absolutely decline to have anything to do with pharmacy or dispensing, and pharmacists who will have nothing to do with prescribing, there is a broad middle zone in which the functions of pharmacist and prescriber overlap. For many medical men not only dispense but even prepare their own drugs or certain of them, whilst many pharmacists not only dispense but also prescribe, that is to say, advise customers whether they should take this or that remedy for symptoms of which they complain.

A Question of Economics.

Many have been the heated arguments both for and against each of these practices, but hitherto it certainly seems to the mere onlooker as though the basis of these arguments had been mainly one of pounds, shillings, and pence as affecting the pocket either of the medical man or the pharmacist. The interest of the patient is far too liable to be lost sight of, and yet this should be kept quite in the forefront all the time if any legislative measures are undertaken to limit in any way prescribing by unqualified persons or dispensing by doctors. It is quite clear that pharmacists would like to see all dispensing transferred to them; it is to their monetary interest that this should be so. It is also to the interests of well-to-do patients, for the turnover and hence the freshness of the drugs is likely to be the greater at the chemist's. In the case of very poor patients, on the other hand, the bottle of medicine is all that is required by them more often than not, and the doctor's practice would disappear almost entirely if dispensing were transferred to the pharmacist. It is not possible to enter into every pro and con here, but, roughly speaking, whereas in the case of rich patients it is better for the medical man to prescribe and the pharmacist to dispense, in that of poor patients, and if it is a question of having either a pharmacist or a medical man to get the medicine from, it must surely be better to have the latter, for the medical man will be better able to detect the small percentage of serious illnesses amongst the host of minor maladies.

Counter Prescribing.

As regards prescribing by chemists and other unqualified persons, it is clearly impossible to prevent this in the present state of the law; but however shrewd a pharmacist may be, he must necessarily be wrong in his diagnosis more often than would be one who has had a skilled medical training. It is true that in a great many cases the pharmacist may be right, or at least he may do no harm; but now and then, if he persistently prescribes for patients whom he is unable to examine thoroughly, and in whom, if he did examine them, he would not be skilled in interpreting all the symptoms, he may sometimes do immeasurable harm either by ordering the wrong treatment or else delaying the attainment of the correct diagnosis until the most curable stage of the malady is past. Prescribing by dispensing pharmacists and other unqualified persons, therefore, is more to be deprecated than is dispensing by medical men.

Unauthorised Repeats.

Some doctors argue that if they send their patients with a prescription to a chemist the patient will return to that chemist to have the prescription made up again and again without coming back to see the doctor. In the majority of cases this is an argument which concerns the pocket of the doctor more than the interests of the patient, and therefore should seldom be given much weight. We contend that throughout all these questions it is the interests of the patient both as regards his pocket and also his health that should be considered first.

A Conference Suggested.

It is a good sign that arrangements have been made for delegates from the British Medical Association to meet delegates from the British Pharmaceutical Conference to discuss these points, and it is to be hoped that the discussion will be broad-minded and free from bitterness, and that it may serve to strengthen the mutual trust that must be developed between the medical practitioner upon the one hand, and the pharmacist upon the other, if prescribing is for the most part to be limited to qualified practitioners and if dispensing is to be delegated to its maximum extent to those best fitted for it—namely, qualified pharmacists.

